# Comparing fixed sampling with minimizer sampling when using *k*-mer indexes to find maximal exact matches

**DOI:** 10.1371/journal.pone.0189960

**Published:** 2018-02-01

**Authors:** Meznah Almutairy, Eric Torng

**Affiliations:** 1 Department of Computer Science and Engineering, Michigan State University, East Lansing, Michigan, United States of America; 2 Department of Computer Science, College of Computer and Information Sciences, Imam Muhammad ibn Saud Islamic University, Riyadh, Saudi Arabia; University of Helsinki, FINLAND

## Abstract

Bioinformatics applications and pipelines increasingly use *k*-mer indexes to search for similar sequences. The major problem with *k*-mer indexes is that they require lots of memory. Sampling is often used to reduce index size and query time. Most applications use one of two major types of sampling: fixed sampling and minimizer sampling. It is well known that fixed sampling will produce a smaller index, typically by roughly a factor of two, whereas it is generally assumed that minimizer sampling will produce faster query times since query *k*-mers can also be sampled. However, no direct comparison of fixed and minimizer sampling has been performed to verify these assumptions. We systematically compare fixed and minimizer sampling using the human genome as our database. We use the resulting *k*-mer indexes for fixed sampling and minimizer sampling to find all maximal exact matches between our database, the human genome, and three separate query sets, the mouse genome, the chimp genome, and an NGS data set. We reach the following conclusions. First, using larger *k*-mers reduces query time for both fixed sampling and minimizer sampling at a cost of requiring more space. If we use the same *k*-mer size for both methods, fixed sampling requires typically half as much space whereas minimizer sampling processes queries only slightly faster. If we are allowed to use any *k*-mer size for each method, then we can choose a *k*-mer size such that fixed sampling both uses less space and processes queries faster than minimizer sampling. The reason is that although minimizer sampling is able to sample query *k*-mers, the number of shared *k*-mer occurrences that must be processed is much larger for minimizer sampling than fixed sampling. In conclusion, we argue that for any application where each shared *k*-mer occurrence must be processed, fixed sampling is the right sampling method.

## Introduction

With each passing year, advances in sequencing technologies, such as ROCHE/454, Illumina/Solexa, and Pacific Biosciences (PacBio), are producing DNA sequences both faster and cheaper. Right now, the average number of sequences generated from one sequencing run is on the order of hundreds of millions to billions. While this explosive growth in DNA datasets yields exciting new possibilities for biologists, the vast size of the datasets also presents significant challenges for many compute-intensive biology applications. These applications include homogenous search [1–4], detection of single nucleotide polymorphisms (SNP) [5–7], mapping cDNA sequences against the corresponding genome [8–10]. sequence assembly [11–13], sequence clustering [14–16], and sequence classification [17–19]. A core operation in all these applications is to search the dataset for sequences that are similar to a given query sequence.

This search process is often sped up by finding shared exact matches (*EM*) of a minimum length *L*. The value of *L* is usually set to ensure all the desired similar sequences are recognized. Depending on the application, the shared *EMs* can be extended to longer *EMs* [17]. In some applications, the *EMs* are extended to maximal exact matches (*MEMs*) [20, 21], and *MEMs* are often extended further to local alignments by allowing mismatches and/or gaps [4, 8, 22, 23].

Finding *EMs*, *MEMs*, and local alignments is often sped up using *k*-mer indexes (*k* < *L*). One of the biggest problems with using *k*-mer indexes is that the size of the index is significantly larger than the underlying database/ datasets. One of the most effective and widely used ways of mitigating *k*-mer index size and query time is to perform sampling, in which we omit some *k*-mer occurrences from the index. A sampling strategy can be classified based on how it chooses *k*-mers. There are two major ways to choose *k*-mers: fixed sampling and minimizer sampling.

It has widely been assumed that fixed sampling produces smaller indexes than minimizer sampling but that minimizer sampling leads to faster query processing than fixed sampling. However, these beliefs have never been empirically verified. In fact, few studies have empirically tested either method on its own [4, 23]. In this paper, we fill this gap by systematically evaluating and comparing fixed sampling and minimizer sampling to assess how these methods perform with respect to index construction time, index size, and query processing time. Specifically, we compare and contrast the construction time, index size, and query processing time required to find all MEMs using both fixed and minimizer sampling.

We start by formalizing the problem of finding *MEMs* between two sequences. Then, we illustrate how *k*-mer indexes are used to find *MEMs*. Next, we formally describe the two *k*-mer sampling methods: fixed sampling and minimize sampling. We highlight the key similarities and differences between these two sampling methods. Finally, we set the comparison framework and conclude with the comparison results.

### The *MEM* enumeration problem

Let *Σ* be a finite ordered alphabet. We focus on the alphabet for nucleotide databases *Σ* = {*A*, *C*, *G*, *T*}. Let *s* be a string over *Σ* of length |*s*|. We use *s*[*i*] to denote the character at position *i* in *s*, for 0 ≤ *i* < |*s*|. We use *s*[0] to denote the first character in string *s*. We use the ordered pair *s*(*i*, *j*) to denote the substring in *s* starting with the character at position *i* and ending with the character at position *j* for 0 ≤ *i* < *j* < |*s*|. We note that substring *s*(*i*, *j*) is also denoted as *s*[*i*..*j*] in some papers, but we only use *s*(*i*, *j*) in this paper.

**Definition 1 *Exact Match* (*EM*)** *For any two strings* *s*_1_ *and* *s*_2_, *a pair of substrings* (*s*_1_(*i*_1_, *j*_1_), *s*_2_(*i*_2_, *j*_2_)) *is an exact match if and only if* *s*_1_(*i*_1_, *j*_1_) = *s*_2_(*i*_2_, *j*_2_). *The length of an exact match is* *j*_1_ − *i*_1_ + 1.

**Definition 2 *Maximal Exact Match* (*MEM*)** *An exact match* (*s*_1_(*i*_1_, *j*_1_), *s*_2_(*i*_2_, *j*_2_)) *is called maximal if* *s*_1_[*i*_1_ − 1] ≠ *s*_2_[*i*_2_ − 1] *and* *s*_1_[*j*_1_ + 1] ≠ *s*_2_[*j*_2_ + 1].

We now formalize the problem of finding *MEMs* between two datasets.

**Definition 3 *MEM Enumeration Problem*** *Given two datasets of sequences* *D*_1_ *and* *D*_2_ *and an integer* *L*, the *MEM* *enumeration problem is to find the set of all* *MEMs* *of length at least* *L* *between all sequences in* *D*_1_ *and all sequences in* *D*2. *We denote this set as* *MEM*(*D*_1_, *D*_2_, *L*). *We use* *MEM*(*L*) *if* *D*_1_ *and* *D*_2_ *are clear from the context*.

We illustrate many of these and later definitions using the following example where *D*_1_ = {*s*_1_} and *D*_2_ = {*s*_2_} and *s*_1_ and *s*_2_ are as follows:

*s*_1_ = *GTAC* *T* *AGG* *CTA* *CTA* *GGGG* with length |*s*_1_| = 18

*s*_2_ = *GTAC* *A* *AGG* *CTA* *CTA* *CTA* *TTTT* with length |*s*_2_| = 21

The two string *s*_1_ and *s*_2_ have two *MEMs* of length at least 6: *AGGCTACTA* = (*s*_1_(5, 13), *s*_2_(5, 13)) with length 9 and *CTACTA* = (*s*_1_(8, 13), *s*_2_(11, 16)) with length 6. Thus, *MEM*({*s*_1_}, {*s*_2_}, 6) = {(*s*_1_(5, 13), *s*_2_(5, 13)), (*s*_1_(8, 13), *s*_2_(11, 16))} whereas *MEM*({*s*_1_}, {*s*_2_}, 8) = {(*s*_1_(5, 13), *s*_2_(5, 13))}.

In this study, we focus on finding *MEMs* of a minimum length *L* between a query sequence and a database of sequences because it is a critical step in searching for local alignments with tools such as NCBI BLAST.

### Using *k*-mer indexes to find *MEMs*

Suffix trees have been the traditional data structure of choice when searching for *MEMs* [20, 24–26]. However, Khiste and Ilie [21] recently showed that *k*-mer indexes use much less space and are more amenable to parallelization compared to suffix trees, at least for larger *MEM*s. This is true despite the development of many new compressed and sparse suffix array data structures [20, 25], Specifically, Khiste and Ilie use fixed sampling to build a memory-efficient *k*-mer index to search for “all *MEMs* of a minimum length 100 between the whole human and mouse genomes”. Their results show that *k*-mer indexes are 2 times faster when both methods are limited to using 4GB of memory. In parallel mode with 12-core machines, *k*-mer indexes are 6 times faster while using 2.6 times less memory. We do note that Khiste and Ilie did not compare their *k*-mer based solution with suffix trees for finding small *MEMs* with minimum lengths as low as 20. It is possible that suffix trees are better for these cases. We focus on finding larger MEMs, so we focus on *k*-mer index solutions to find MEMs.

Finding *MEMs* with a minimum length *L* is often sped up using a *k*-mer index, *k* ≤ *L*, at the cost of additional space. A *k*-mer index supports quickly finding *EMs* of length *k* which are also known as shared *k*-mers. We typically extend these shared *k*-mers in two stages. In the first stage, we try to extend every shared *k*-mer into an *EM* of length *L* ≥ *k*. If the first stage is successful, we then further extend the match into an *MEM*. In the context of searching for local alignments, every *MEM* is extended even further by allowing mismatches and/or gaps.

It is possible to skip the first extension step and build an *L*-mer index to find all shared *L*-mers. However, due to technical limitations and the huge memory requirements necessary for building an *L*-mer index for *L* > 32, it is common to build the index using *k* ≤ 32 < *L* [4, 17, 21, 23].

We typically work with a *k*-mer index as follows. We save the list of *k*-mers present in the database and we refer to this list as dictionary. For each saved *k*-mer, we save some of its occurrences into a list. When given a query sequence, we extract *k*-mers from the query, see if that *k*-mer appears in the dictionary, and find the corresponding shared *k*-mers by using the stored list of occurrences.

We describe this search process more precisely as follows.

**Definition 4 (*k*-mer and *k*-mer occurrence)** *Consider any length* *k* *substring* *s*(*j* − *k* + 1, *j*) *of string* *s* *where* *k* − 1 ≤ *j* ≤ *s* − 1. *We call that substring a* *k*-*mer and more concisely represent this* *k*-*mer occurrence using the ordered pair* (*s*, *j*).

**Definition 5 (Shared *k*-mers and shared *k*-mer occurrences)** *Consider any two strings* *s*_1_ *and* *s*_2_ *that have an exact match* (*s*_1_(*i*_1_, *j*_1_), *s*_2_(*i*_2_, *j*_2_)) *of length* *k*. *We call the common substring* *s*_1_(*i*_1_, *j*_1_) (*equivalently* *s*_2_(*i*_2_, *j*_*w*_)) *a shared* *k*-*mer and more concisely represent the corresponding shared* *k*-*mer occurrence using the quadruple* (*s*_1_, *j*_1_, *s*_2_, *j*_2_).

Any string *s* of length |*s*| contains exactly |*s*| − *k* + 1 *k*-mer occurrences. Using our previous example with *k* = 3, *s*_1_ and *s*_2_ have 16 and 19 3-mer occurrences, respectively. Furthermore, *s*_1_ and *s*_2_ have exactly seven shared 3-mers: *ACT*, *AGG*, *CTA*, *GCT*, *GGC*, *GTA*, and *TAC*. These shared 3-mers result in 24 different shared 3-mer occurrences as follows. *GCT*, *GGC*, and *GTA* appear exactly once in *s*_1_ and *s*_2_, and thus each of them has exactly one shared 3-mer occurrence: (*s*_1_, 9, *s*_2_, 9), (*s*_1_, 8, *s*_2_, 8), and (*s*_1_, 2, *s*_2_, 2). The 3-mer *AGG* occurs 2 times in *s*_1_ and 1 time in *s*_2_, and thus *AGG* is part of two different shared 3-mer occurrences: (*s*_1_, 5, *s*_2_, 7) and (*s*_1_, 15, *s*_2_, 7). Since shared 3-mer *ACT* occurs 2 times in both *s*_1_ and in *s*_2_, the shared 3-mer *ACT* is part of 2 × 2 different shared 3-mer occurrences: (*s*_1_, 4, *s*_2_, 12), (*s*_1_, 4, *s*_2_, 15), (*s*_1_, 12, *s*_2_, 12), and (*s*_1_, 12, *s*_2_, 15). Similarly, *TAC* occurs 2 times in *s*_1_ and 3 times in *s*_2_, so shared 3-mer *TAC* is part of 3 × 2 different shared 3-mer occurrences: (*s*_1_, 3, *s*_2_, 3), (*s*_1_, 3, *s*_2_, 11), (*s*_1_, 3, *s*_2_, 14), (*s*_1_, 11, *s*_2_, 3), (*s*_1_, 11, *s*_2_, 11), and (*s*_1_, 11, *s*_2_, 14). Finally, *CTA* occurs 3 times in *s*_1_ and 3 times in *s*_2_, leading to 3 × 3 different shared 3-mer occurrences: (*s*_1_, 5, *s*_2_, 10), (*s*_1_, 5, *s*_2_, 13), (*s*_1_, 5, *s*_2_, 16), (*s*_1_, 10, *s*_2_, 10), (*s*_1_, 10, *s*_2_, 13), (*s*_1_, 10, *s*_2_, 16), (*s*_1_, 13, *s*_2_, 10), (*s*_1_, 13, *s*_2_, 13), and (*s*_1_, 13, *s*_2_, 16).

Since *k* ≤ *L*, it is possible that a shared *k*-mer occurrence is not part of an *MEM* of length at least *L*; we call such a shared *k*-mer occurrence a **false positive**. In general, decreasing the value of *k* increases the chance that a shared *k*-mer occurrence is a false positive.

Using the above 24 shared 3-mers occurrences and assuming *L* = 6, the *MEM* = *AGGCTACTA* can be found by extending any of the following seven 3-mer occurrences: (*s*_1_, 7, *s*_2_, 7), (*s*_1_, 8, *s*_2_, 8), (*s*_1_, 9, *s*_2_, 9), (*s*_1_, 10, *s*_2_, 10), (*s*_1_, 11, *s*_2_, 11), (*s*_1_, 12, *s*_2_, 12), or (*s*_1_, 13, *s*_2_, 13). Similarly the *MEM* = *CTACTA* can be found by extending any of the following four 3-mer occurrences: (*s*_1_, 10, *s*_2_, 13), (*s*_1_, 11, *s*_2_, 14), (*s*_1_, 12, *s*_2_, 15), or (*s*_1_, 13, *s*_2_, 16). The remaining twelve shared 3-mer occurrences are false positives. If *L* = 8, then the seven shared 3-mer occurrences that can be extended to *AGGCTACTA* are not false positives. The remaining sixteen shared 3-mer occurrences are false positives.

Every *EM* of length *L* has *L* − *k* + 1 shared *k*-mer occurrences. Finding and extending one of these shared *k*-mer occurrences is sufficient for finding that *EM*. Therefore, when building *k*-mer indexes, we can store a sampled subset of *k*-mer occurrences in the index and still find every possible *MEM* of length at least *L*. With sampling, we not only reduce the index’s memory requirements, we also reduce query time by not discovering the same *MEM* multiple times. Sampling, therefore, is a very effective method for improving a *k*-mer index’s efficiency (reducing construction time, space, and query time).

### Fixed sampling versus minimizer sampling

In bioinformatics, two sampling methods are commonly used to build *k*-mer indexes: **fixed sampling** [4, 21] and **minimizer sampling** [17, 23]. To ensure that a *k*-mer index achieves 100% sensitivity which means that it finds all shared *MEMs* of length at least *L*, both methods ensure that within every *MEM* of length at least *L*, at least one *k*-mer occurrence is saved to the index. We now define both sampling methods comparing and contrasting their relative strengths and weaknesses.

Fixed sampling is a simple greedy sampling strategy that minimizes the number of *k*-mer occurrences stored in the index. The goal is to ensure we choose one complete *k*-mer from every possible substring of length *L* from each database sequence *s*. For example, we must choose one *k*-mer from *s*(0, *L* − 1) to store in the index; we greedily choose the *k*-mer that ends at *s*[*L* − 1] since it not only covers this substring but also the next *L* − *k* − 1 substrings up to but not including *s*(*L* − *k* + 1, 2*L* − *k*). To cover the substring *s*(*L* − *k* + 1, 2*L* − *k*), we again greedily choose the *k*-mer that ends at *s*[2*L* − *k*] since it again covers the next *L* − *k* − 1 substrings. In general, the *j*th *k*-mer occurrence that we sample ends at position *L* − 1 + (*j* − 1)*w* where *w* = *L* − *k* + 1. We typically refer to *w* = *L* − *k* + 1 as our sampling step or sampling window for fixed sampling. During the query phase, we extract every *k*-mer from the query sequence *q* to search for shared *k*-mer occurrences. Since every *k*-mer is extracted from *q*, if *s* and *q* have an *MEM* of length at least *L*, then some shared *k*-mer from that *MEM* will be in the *k*-mer index and the *MEM* can be recovered. Fixed sampling has several advantages. First, it is very fast to construct the index requiring no *k*-mer comparisons and skipping over significant portions of the database sequence *s*. Second, it stores the minimum possible number of *k*-mer occurrences in the index to guarantee 100% sensitivity and thus minimizes index size. The disadvantage is that all *k*-mers from the query sequence need to be processed which may slow query time.

Minimizer sampling uses a more sophisticated sampling strategy that allows sampling of both the database sequence *s* and the query sequence *q*. We must again choose one *k*-mer from *s*(0, *L* − 1) to store in the index. This time, we choose to store the **minimum** *k*-mer from *s*(0, *L* − 1) in our index where we order substrings in some canonical order. For simplicity, one can use the alphabetical order where *A* < *C* < *T* < *G* which implies *AAA* < *AGA* < *AGG* < *TAA*. Roberts *et al.* recognized using an alphabetical ordering can have issues, particularly in low complexity regions where there may be repeats of characters or short substrings. To handle the low complexity regions, Roberts *et al.* write “In general, we want to devise our ordering to increase the chance of rare *k*-mers being minimizers.” For example, Robert *et al.* proposed using *C* < *A* < *T* < *G* in odd numbered bases and the reverse ordering in even-numbered bases [23]. Alternatively, Wood *et al.* [17] suggest using the exclusive-or (XOR) operation to scramble the standard ordering of each *k*-mer before comparing the *k*-mers to each other using lexicographical ordering. Once an ordering is established, we process each length *L* substring of *s* (equivalently each window of *w* = *L* − *k* + 1 *k*-mers) in turn storing the minimum *k*-mer occurrence in the index. The first view focusing on substrings of length *L* seems more intuitive; the second view focusing on windows of *w* *k*-mers is useful when predicting the expected size reduction from using minimizer sampling. We note a few things. First, there may be multiple occurrences of a minimum *k*-mer within a length *L* substring; in this case for the original minimizer algorithm but not the one we focus on in this paper, each occurrence is stored in the index. Second, minimizer sampling is likely to store more occurrences than fixed sampling as it does not maximize the distance between *k*-mer occurrences stored in the index. Third, the time to construct the index is a bit slower as more work is done for each window including comparing different *k*-mers. The advantage that minimizer has comes at query time. Rather than choosing all *k*-mers from query *q*, minimizer sampling applies the same sampling strategy to *q*. That is, we consider every substring of length *L* and choose only the minimum *k*-mers from within each length *L* substring to consider for extension. Since both the query and each database string extract the minimum *k*-mer(s) from every substring of length *L*, if there is an *EM* of length *L*, the same minimum *k*-mer will be extracted and then extended into the *EM* and then *MEM*. In summary, minimizer sampling requires a bit more time to construct its index though it can do so in linear time and builds a larger index than fixed sampling, but it processes fewer *k*-mers from the query sequence and thus may have faster query processing times.

We illustrate the two algorithms using our previous example where we use *k* = 3 and *L* = 8 so *w* = *L* − *k* + 1 = 6, and we use *D*_1_ = {*s*_1_} as our database and *D*_2_ = {*s*_2_} as our query dataset. The goal is to return *MEM*({*s*_1_}, {*s*_2_}, 8). With fixed sampling, we store the 3-mer *AGG* with its occurrence (*s*_1_, 7) and the 3-mer *CTA* and its occurrence (*s*_1_, 13) in the 3-mer index.

All 19 3-mers from *s*_2_ are extracted with both *AGG* and *CTA* being shared 3-mers. Since *CTA* occurs three times in *s*_2_, we consider four shared 3-mer occurrences for extension: (*s*_1_, 7, *s*_2_, 7), (*s*_1_, 13, *s*_2_, 10), (*s*_1_, 13, *s*_2_, 13), (*s*_1_, 13, *s*_2_, 16). The first and third can be extended to the same *MEM* of length 9 whereas the other two cannot be extended to a length 8 *MEM* and thus are false positives.

With minimizer sampling, we store three 3-mer occurrences to the index, two with *ACT* and one with *AGG*: (*s*_1_, 4), (*s*_1_, 7), and (*s*_1_, 12). Minimizer sampling is also applied to the query *s*_2_ and three 3-mer occurrences are chosen to test for extension, two with *ACT* and one with *AAG*: (*s*_2_, 7), (*s*_2_, 12), and (*s*_2_, 15). The only shared 3-mer is *ACT*, and since *ACT* appears twice in both sequences, we consider four shared 3-mer occurrences for extension: (*s*_1_, 4, *s*_2_, 12), (*s*_1_, 4, *s*_2_, 15), (*s*_1_, 12, *s*_2_, 12), and (*s*_1_, 12, *s*_2_, 15). Only (*s*_1_, 12, *s*_2_, 12) can be extended to an *MEM* of length at least 8; the other three shared *k*-mer occurrences are false positives.

There are two possible optimizations that can be used to improve the performance of minimizer sampling. The first one is to avoid using lexicographical ordering. The second one is not to sample duplicate minimizers. In this paper, we study the effects of each optimization individually and combined. To avoid lexicographical ordering, we use the randomization method suggested by Wood *et al.* [17]. To prevent sampling duplicate minimizers, we use the robust winnowing method proposed by Schleimer *et al.* [27], but only apply robust winnowing to the index, not to the query sequences. We sample all minimizers from a query sequence window. In this scenario, the correct minimizer occurrence matches are guaranteed to be found. We finally apply both methods together to test the effectiveness of using both optimizations simultaneously.

### Problem statement and overall aims

It has widely been assumed that fixed sampling produces smaller indexes than minimizer sampling but that minimizer sampling leads to faster query processing than fixed sampling. For example, Roberts *et al.* [23] highlight the importance of sampling *k*-mers at query time during the search procedure saying that “the procedure would still be more efficient if we could compare only a fraction of the *k*-mers in *T* to the database” where *T* is their notation for a set of query strings. However, these beliefs have never been empirically verified. In fact, few studies have empirically tested either method on its own [4, 23].

In this paper, we fill this gap by **systematically evaluating and comparing fixed sampling and minimizer sampling to assess how these methods perform with respect to index construction time, index size, and query processing time**. Specifically, we compare and contrast the construction time, index size, and query processing time required to solve the *MEM* Enumeration Problem using both fixed and minimizer sampling. We use the human genome as our database and the mouse genome, the chimp genome, and an NGS data set as our three query sets. Our goal is to provide guidance to developers of *k*-mer-based bioinformatics tools so that they can choose the best method for their application.

Our main contributions are the following: First, we systematically compare fixed sampling with minimizer sampling using real biological datasets to assess how well they find all *MEMs* with respect to index construction time, index size, and query processing time. Our results show that if we use the same *k*-mer size, minimizer sampling is only slightly faster than fixed sampling, despite sampling from the query sequence. If we are allowed to use any *k*-mer size for each method, then we can choose a *k*-mer size such that fixed sampling both uses less space and processes queries faster than minimizer sampling. Second, we evaluate the impact of the *k* value on the effectiveness of fixed and minimizer sampling methods to find all *MEMs*. Previous studies usually focus on only one value of *k*. We show that the value of *k* has a significant impact on a sampling method’s index size and query processing time. When the value of *k* decreases, fewer *k*-mer occurrences are saved resulting in smaller indexes. However, when the value of *k* increases, the index processes queries much faster. On average, the reduction in query times for all query sets when *k* = 32 compared to *k* = 12 is 37 and 136 times faster for fixed sampling and minimizer sampling, respectively.

### Related work

Over the last decade, there has been dramatic increase in the use of *k*-mer indexes in biological applications and pipelines to accelerate the search for *EMs*, *MEMs*, or local alignments. Since *k*-mer indexes require a lot of memory compared to the underlying databases, sampling has been widely used to reduce index size. Two primary sampling methods have been proposed in the literature for building *k*-mer indexes: fixed sampling [4, 21] and minimizer sampling [23, 28, 29]. Fixed sampling is the dominant sampling method in practice since it is used in indexed BLAST [4]. Minimizer sampling was proposed in 2004 by Roberts *et al.* but was largely ignored for many years until roughly 2012 when papers began using the minimizer sampling concept for a wide variety of bioinformatics applications [17, 28–34]. Recently, tools have been developed that use minimizer sampling to reduce *k*-mer index size and increase efficiency [28, 29]; both tools map long reads (10kb or longer) that are produced by new sequencing technologies (Single Molecule Real Time and Oxford Nanopore Technologies) against large reference databases.

Despite the fact that both sampling methods have existed for some time, no previous work has compared fixed sampling with minimizer sampling to determine their relative benefits and weaknesses with respect to building *k*-mer indexes to search for *EMs*, *MEMs*, or local alignments. Specifically, Roberts *et al.* did not compare to fixed sampling when they presented minimizer sampling; they compared instead to no sampling [23]. Morgulis *et al.* did not compare to minimizer sampling when they presented their work on fixed sampling in indexed BLAST [4]. Khiste and Ilie only consider fixed sampling when analyzing *k*-mer based indexes [21]. Li as well as Jain *et al.* did not consider fixed sampling in their work [28, 29]. We fill in this gap by carefully comparing these two strategies to determine how well they perform for this important problem.

Schleimer *et al.* [27] independently introduced minimizer sampling calling it winnowing sampling. While winnowing is identical to minimizer, winnowing has been studied and used for different problems and domains than minimizer. Minimizer has been used with biological datasets to solve the problem of searching for local alignments whereas winnowing has been used with text document datasets to solve the problems of *k*-mer counting and ranking to detect document plagiarism. A key difference between minimizer and winnowing sampling is that minimizer sampling requires saving sampled *k*-mer positions whereas winnowing sampling does not. This is because in *MEM* and *HSLA* search problems, the positions define anchor points to compare sequences whereas in *k*-mer counting and ranking problems, only the number of occurrences is used to estimate the similarity between two documents. Similar to Roberts *et al.*, Schleimer*et al.* observed that in low complexity regions (or low-entropy strings in text mining literature), a *k*-mer might occur more than once and all of its occurrences are sampled. Schleimer addressed this problem by not sampling duplicate *k*-mers in both the indexing and the querying phases; they call this version robust winnowing. Schleimer *et al.* did not compare the performance of winnowing and robust winnowing.

We now summarize some other applications which have used *k*-mer sampling in bioinformatics. We emphasize that these all differ from our application and our not directly comparable. We start with sequence assembly. Ye *et al.* [30] proposed using sampled *k*-mers, instead of all *k*-mers, to reduce the memory requirements for De Bruijn graph (DBG) based assemblers. Ye *et al.* used fixed sampling to sample *k*-mers; the penalty is that links or edges between *k*-mers are longer and slightly more complex. Ye *et al.* report that fixed sampling with step *w* reduces their dictionary by roughly 1/*w* compared to tools that use a full list of *k*-mers [35–37]. Ye *et al.* note the existence of minimizer sampling and express interest in comparing minimizer sampling to fixed sampling in future work but did not compare the two in their work. In genome assembly, we only use the the dictionary (the list of *k*-mers); we do not use the lists of *k*-mer occurrences. In other applications, where we use the lists of *k*-mer occurrences, the size of these lists is the dominant factor in index size. Therefore it is important to understand how fixed and minimizer sampling affect both the number of *k*-mers and the number of *k*-mer occurrences. Li *et al.* [38] and Movahedi *et al.* [31] both proposed disk-based DBG assemblers to avoid loading the whole graph into RAM. They load small segments of the graph incrementally, and complete the assembly in this fashion. Since completing the assembly requires identifying adjacent *EMs*, both papers use minimizer sampling as a hashing mechanism to find adjacent *EMs* and group them into the same segment. We do not focus on these applications for two reasons. First, our goal is to focus on applications that use *k*-mer indexes in RAM, which means that index size is critical. Second, minimizer sampling, in the above context, is used as a hashing function to minimize disk I/O operations rather than reducing the list of *k*-mers.

In the *k*-mer counting problem, the task is to build a histogram of occurrences of every *k*-mer in a given data set where *k* is relatively large (*k* > 20) and it is infeasible to list all *k*-mers in RAM. Similar to disk based DBG assemblers, minimizer sampling is used to select *m*-mers (*m* < *k*) from every *k*-mer. These *m*-mers are later used to reduce disk I/O operations in disk based counting *k*-mers tools such as MSPKmerCounter [33] and KMC2 [34]. Again, this problem is significantly different than our motivating problems which are searching for *MEMs* and *HSLAs*. In *MEM* and *HSLA* search problems, the location of sampled *k*-mers is important.

In metagenomic sequence classification, Kraken [17] uses the idea of minimizer to accelerate the classification process in large data sets. Kraken starts with creating a database that contains entries of an *L*-mer and the lowest common ancestor *LCA* of all organisms whose genomes contain that *L*-mer. When a query sequence is given, Kraken searches the database for each *L*-mer in a sequence, and then uses the resulting set of *LCA* taxa to determine an appropriate label for the sequence. To find all *L*-mers effectively, Kraken builds a *k*-mer index, (*k* < *L*) where each *k*-mer is associated with all *L*-mers containing this *k*-mer as a its minimizer. Since a simple lexicographical ordering of *k*-mers can be biased to sample more minimizers over low-complexity regains, Kraken uses the exclusive-or (XOR) operation to scramble the standard ordering of each *k*-mer’s canonical representation before comparing the *k*-mers to each other using lexicographical ordering.

Recently Orenstein *et al.* [39] proposed a new *k*-mer sampling method called *DOCKS*. For a given data set, *L* and *k* (*k* < *L*), the task is to find the minimum-size set of *k*-mers such that for every *L*-mer in the data set, at least one *k*-mer is in this *L*-mer. They show that *DOCKS* sampling results in a much smaller set compared to *minimizer* sampling. It will be interesting to compare fixed sampling and minimizer sampling with *DOCKS* in future work.

In this study we focus on *k*-mer indexes that find all shared *EMs* of length *L*. All indexes are built with *w* = *L* − *k* + 1 and thus achieve 100% sensitivity. Almutairy and Torng [40] proposed to study the impact of the sampling parameter *w* on the effectiveness of *k*-mer indexes to find all highly similar local alignments (*HSLAs*) using NCBI BLAST. In their paper, *k*-mer indexes are built using the default BLAST setting where *k* = 12 and fixed sampling. To study the impact of *w*, they build indexes with a wide range of *w* values where *w* > *L* − *k* + 1. They compared the indexes’ effectiveness with respect to baseline indexes; which are indexes created using *w* = *L* − *k* + 1. They show very large *w* can still achieve high sensitivity; that is, even with very large *w*, fixed sampling can find almost all *HSLAs*. This is slightly different than our study where we focus on finding all *MEMs* and not all *HSLAs*. Our results about the impact of the value of *k* on fixed sampling would enhance their findings since both *k* and *w* play a major role in *k*-mer index efficiency.

## Materials and methods

We represent nucleotides using two bits and store *k*-mers for *k* ≤ 32 in a 64-bit block. All indexes are saved as hash tables where a key is a *k*-mer and its value is a pointer to that *k*-mer’s list of occurrences. We store each *k*-mer’s list of occurrences in a set data structure; each occurrence is an ordered pair of 64-bit positive integers (*s*, *j*) where *s* is a sequence ID and *j* is the ending position of this *k*-mer occurrence in *s*.

### Sampling methods

We compare fixed sampling and minimizer sampling. Minimizer sampling can be improved using two different optimizations: randomized ordering and duplicate minimizer removal. We list all possible combinations of our two optimizations for minimizer sampling in Table 1.

**Table 1:** Possible minimizer sampling versions based on the optimization techniques.

Duplicate handling \Ordering schema	Lexicographical	Randomized
Sample all duplicate minimizers	*min*_*lex*,*many*_	*min*_*rand*,*many*_
Remove duplicate minimizers	*min*_*lex*,*one*_	*min*_*rand*,*one*_

Using both optimizations will result in the most effective minimizer method *min*_*rand*,*one*_. Thus, we compare *min*_*rand*,*one*_ with fixed sampling (*fix*) to test the effectiveness of the two major sampling methods. We compare *min*_*lex*,*one*_ with min *min*_*rand*,*one*_ to determine how much effect the randomization optimization has, and we compare *min*_*rand*,*many*_ with min *min*_*rand*,*one*_ to determine how effective duplicate removal is. We will not consider *min*_*lex*,*many*_ in any comparison since it is the worst version of minimizer and known to be inefficient. Next, we formally describe each sampling method. Note that we choose parameters that ensure we achieve 100% sensitivity which means we will find all *MEM*s.

In fixed sampling (*fix*), as we described earlier, we build the *k*-mer index for a database of sequences by sampling from every database sequence *s* the *k*-mer occurrences ending at positions *L* − 1 + (*j* − 1)*w* where *w* = *L* − *k* + 1 and 0 ≤ *j* ≤ ⌊(|*s*| − *L* + 1)/*w*⌋. We refer to *w* = *L* − *k* + 1 as our sampling step. During the query phase, we extract all *k*-mer occurrences from each query sequence *q* and consider them for extension. Since every *k*-mer is extracted from *q*, if a database sequence *s* and *q* have an *MEM* of length at least *L*, then some shared *k*-mer from that *MEM* will be in the *k*-mer index and the *MEM* can be recovered.

In standard minimizer sampling without any optimization (*min*_*lex*,*many*_), for every substring of length *L* in a database sequence *s*, we store the minimum *k*-mer occurrence in our index; if there is more than one minimum *k*-mer within any length *L* substring, all minimum *k*-mer occurrences are stored. We use the normal lexicographical ordering where *A* < *C* < *T* < *G* to define minimum *k*-mers in our work. Unlike fixed sampling, during the query phase, we use the same sampling for a query sequence *q*. That is, we consider only the minimum *k*-mers from each substring of length *L* in *q* for extension. Since both the query and each database string extract the minimum *k*-mer(s) from every substring of length *L*, if there is an *EM* of length *L*, the same minimum *k*-mer will be extracted and then extended into the *EM* and then *MEM*.

We find minimum *k*-mers from *s* and *q* using a linear time sliding window approach proposed by Smith [41]. The basic idea is to store *k*-mers in a double-ended queue or deque that allows fast insertion and deletion at both its beginning and its end. We maintain the following invariant for the deque. The deque contains *k*-mers from the eligible window sorted in two ways: by *k*-mer value where the smallest *k*-mer value is at the front and the largest *k*-mer value is at the rear and by location where the oldest *k*-mer is at the front and the newest *k*-mer is at the rear. The basic reason for these invariants is that any *k*-mer that is larger and further to the left of the most recent *k*-mer will never be a minimizer in any future windows since this new *k*-mer will be in any such windows and has smaller value. We maintain this invariant in two ways. First, when we add the new *k*-mer that is in this window, we compare it to the *k*-mer at the rear of the deque and remove that rear *k*-mer if it is larger than the current *k*-mer. We continue in this fashion until we find a *k*-mer smaller than the new *k*-mer or the deque is empty and we add the new *k*-mer to the deque at the rear of the deque. Second, we find the minimizer by pulling the first element from the deque. However, we must first verify this *k*-mer is still in the current window. If not, we remove it from the deque and use the next *k*-mer as the minimizer. We can see this takes linear time using the following charging scheme where we charge each comparison to the item that was deleted or inserted into the deque. Each item is charged twice, once for when it is inserted, and once for when it is deleted. Thus, the total number of comparisons is linear in the number of *k*-mers processed.

Now, we describe how to apply optimizations to reduce the number of *k*-mers sampled from the database which leads to a significant speedup of query time. The first optimization is to use randomized ordering instead of lexicographical ordering. To do this, we first create a random *k*-mer mask by uniformly selecting *k* letters from *A*, *C*, *G*, or *T* in each position. We then view a k-mer as a 2k bit string. For any k-mer or equivalently 2k bit string, we create a new scrambled 2k bit string by doing an exclusive-or (XOR) operation between every bit of the 2k bit string and the 2k bit mask. We then sort all the scrambled 2k bit strings to identify a minimizer. For example, the bit string for the 4-mer *AACC* is 00000101 using lexicographical ordering, where *A* = 00, *C* = 01, *G* = 10, and *T* = 11. Let the random 4-mer mask be *CGAT* which is equivalent to the 8-bit string 01100011. After applying the XOR operation between *AACC* (00000101) and *CGAT* (01100011), the resulting scrambled bit string for *AACC* is 01100110. We refer to minimizer sampling that uses this randomized ordering as *min*_*rand*_.

The second optimization prevents sampling duplicate minimizers in the indexing phase. There are two occasions where standard minimizer stores duplicate minimizers in the index. The first occurs when the current minimizer is not part of the next window and we must examine all *w* = *L* − *k* + 1 *k*-mers in that window. If we find multiple minimizers, all are stored in the index using the standard minimizer sampling strategy. To apply the duplicate removal optimization, we store only the rightmost minimizer in the index. The second possibility for storing duplicate minimizers occurs when the current minimizer for the previous window still lies within the next window and is identical to the one new *k*-mer for that window. In this scenario, to remove duplicates, we do not store this duplicate copy at this time in the index. However, we do track its position so that if no new minimizer is found before the current minimizer moves out of the current window, we can use this *k*-mer to replace the current minimizer at that time and still do only one comparison for that window. At that time, we would have to store this minimizer in the index if it is the minimizer of that window. We refer to minimizer sampling that uses duplicate removal as *min*_*one*_.

### Indexing and querying

In the indexing phase, we create a *k*-mer index for a given database and sampling method as follows. We sample *k*-mers and their occurrences from each sequence based on the selected sampling method (*fix*, *min*_*rand*,*one*_, *min*_*rand*,*many*_, and *min*_*lex*,*one*_). We save the sampled *k*-mers into the index dictionary, and for each *k*-mer occurrence, we update the corresponding list of *k*-mer occurrences.

We then proceed to the querying phase where we sequentially process each query sequence. If we use fixed sampling (*fix*), we extract all *k*-mer occurrences from query sequence *q*. For all minimizer methods *min*_*rand*,*one*_, *min*_*rand*,*many*_, and *min*_*lex*,*one*_, we extract the minimum *k*-mer occurrences including duplicates from each window in *q*.

Once we extract the *k*-mer occurrences from *q*, we use the index to find shared *k*-mer occurrences and then *MEMs* as follows. For every *k*-mer occurrence in *q*, we check if the *k*-mer is in the index dictionary. If the *k*-mer is found, then we use the *k*-mer’s associated list of occurrences to find all shared *k*-mer occurrences between *q* and the database of sequences.

We perform this search in a manner similar to NCBI BLAST with the goal of minimizing the number of database read operations. Specifically, we group the shared *k*-mer occurrences between *q* and *DB* by database sequence ID *s*. For the list of *k*-mer occurrences shared between *q* and *s*, we sort them in alphabetical order and then positional order. We store this information in a hash table with key *s* where the hash table entries are pointers to the sorted lists of shared *k*-mer occurrences. We then read in each relevant database sequence *s* exactly once and process all the corresponding shared *k*-mers in alphabetical order of *k*-mer.

For every query sequence *q* and a database sequence *s*, we report any shared *k*-mer occurrence that can be extended to length at least *L* as an *MEM*. Our extension method is similar to that of Khiste and Ilie [21]. Before we try to extend a shared *k*-mer occurrence, we first check if it is contained within our list of discovered *MEMs* which is, of course, initially empty. If so, we skip this shared *k*-mer occurrence and move on to the next one. If not, then we try to extend the *k*-mer in both directions to see if it is part of an *MEM* with length at least *L*. If the extension succeeds, we add the new *MEM* to our list of discovered *MEMs*. If the extension fails, we report this shared *k*-mer occurrence as a false positive. This ensures we only extend one shared *k*-mer occurrence within any *MEM*.

We check if a shared *k*-mer occurrence is part of a discovered *MEM* using the following properties. A shared *k*-mer occurrence $\left(q,j_{q}^{\prime },s,j_{s}^{\prime }\right)$ is part of a shared *MEM* *MEM* = (*q*(*i*_*q*_, *j*_*q*_), *s*(*i*_*s*_, *j*_*s*_)) if the following conditions hold: (1) $i_{q}\leq j_{q}^{\prime }-k+1<j_{q}^{\prime }\leq j_{q}$, (2), $i_{s}\leq j_{s}^{\prime }-k+1<j_{s}^{\prime }\leq j_{s}$, and (3) $\left(j_{q}^{\prime }-k+1\right)-i_{q}=\left(j_{s}^{\prime }-k+1\right)-i_{s}$. Checking these conditions can be done in constant time per discovered *MEM*, and typically the number of discovered *MEM*s per pair of sequences *q* and *S* is small, so this verification step typically takes constant time.

### Experimental setting and evaluation metics

#### Database and query sets

We consider only nucleotide datasets. Our database is the human genome. We use three query sets: the mouse genome, the chimp genome and an NGS dataset. All the datasets are publicly available. The genome datasets can be downloaded from UCSC (http://hgdownload.cse.ucsc.edu). The NGS dataset can be downloaded from Sequence Read Archive (SRA) on the NCBI website (https://www.ncbi.nlm.nih.gov/sra) Table 2 describes each dataset used. According to Koning *et al.* [42], two third of the human genome consists of repetitive sequences. It is also known that the mouse genome contains many repeats too. [4, 43]. It is unclear if this is the case for the chimp and NGS datasets.

**Table 2:** datasets used for testing.

Datasets	Size (Mbp)	#Seq.	Type	# Processed Seq.
*Homo sapiens* (Human)	3137	93	Database	2,897,341
*Mus musculus* (Mouse)	2731	66	Query set	5,306
*Pan troglodytes* (Chimp)	3218	24,132	Query set	5,818
*SRA:SRR003161* (NGS)	788.5	1,376,701	Query set	2,792

The database set is only one large set. The query sets are partitioned into 1000 small query sets where each small set has the indicated number of processed sequences (except the last set may have fewer sequences). The processed sequences are of length 1000 (except the last processed sequence for every sequence may be shorter).

Performing the queries using each query set directly would require lots of computing power, memory and time. For example, the number of *MEMs* of length at least 50 is more than two billion when we compare the human and the chimp genomes. Khiste and Ilie [21] proposed to store the *MEM*s in files based on their starting positions in the query genome. Later, each file is sorted, duplicates are removed, and MEMs are output in the right order. In our case, we pre-process all the datasets by dividing every sequence into non-overlapping sequences of length 1000. Therefore, we process more but smaller sequences while keeping the list of *MEMs* for each sequence manageable in RAM. This pre-processing also supports parallelization of queries on MSU’s High Performance Computing Cluster. The number of resulting sequences is shown in Table 2. We also only save letters in {*A*, *C*, *G*, *T*}; that is, ambiguous characters are removed. For each pre-processed query set, we partition the set into 1000 query sets of equal size (except the last set may be slightly smaller). We recognize that we may not be able to find *MEMs* that extend across the pre-processed sequences, but this should not significantly change our results.

For each of our pre-processed query sets, we compute the actual number of MEMs between that query set and the pre-processed human genome for both choices of *L*. These results are shown in Table 3.

**Table 3:** The number of *MEM*s for each query set and the human genome for both choices of *L* given our pre-processing into sequences of length 1000.

L	Mouse	Chimp	NGS
50	838,857,328	2,077,183,744	940,731
100	428,609	101,868,611	457,512

#### Index parameters and metrics

We study the impact of the sampling methods on the *k*-mer index creation phase. We consider the following sampling methods *fix*, *min*_*rand*,*one*_, *min*_*lex*,*one*_, and *min*_*rand*,*many*_. We use the index to find all *MEMs* of length at least *L* where *L* ∈ {50, 100}. For each sampling method, we create a set of indexes for *k* ∈ [12, 32]. We consider *L* = 50 and *L* = 100, because both are frequently used in biological applications that compare the mouse and chimp genomes to the human genome [20, 21] or map an NGS dataset against the human genome [8, 22].

For each index, we report the dictionary size, lists size and the total index size which is the sum of dictionary and lists sizes. The dictionary size is measured by counting the number of *k*-mers. The lists size is measured by counting the number of *k*-mers occurrences in all lists. We also report the index construction time.

#### Querying parameters and metrics

The index is used to find all *MEMs* of length at least *L* where *L* ∈ {50, 100}. For *L* and for each sampling method, we used a set of *k*-mer indexes where *k* ∈ {12, 16, 20, 24, 28, 32}. The total number of indexes considered is 2 × 3 × 6 = 48 indexes. All the indexes give the same final results, namely all *MEMs* of length at least *L*.

For each query phase, we report the time and the number of “false positives”. The number of false positives for a query set is the number of shared *k*-mer occurrences that failed to be extended to a *MEM* of length at least *L*. Recall that we partition each query set into 1000 query set partitions. The reported time is the sum of times that an index needs to answer all queries in all query set partitions. Likewise, the number of false positives for a query set is the sum of the number false positives for all queries in all query set partitions..

#### System specification/configuration

We run the experiments on a cluster that runs the Community Enterprise Operating System (CentOS) 6.6. The cluster has 24 nodes where each node has two 2.5Ghz 10-core Intel Xeon E5-2670v2 processors, 256 GB RAM, and 500 GB local disk.

## Results and discussion

### Index size and index construction time

**Fig 1:**
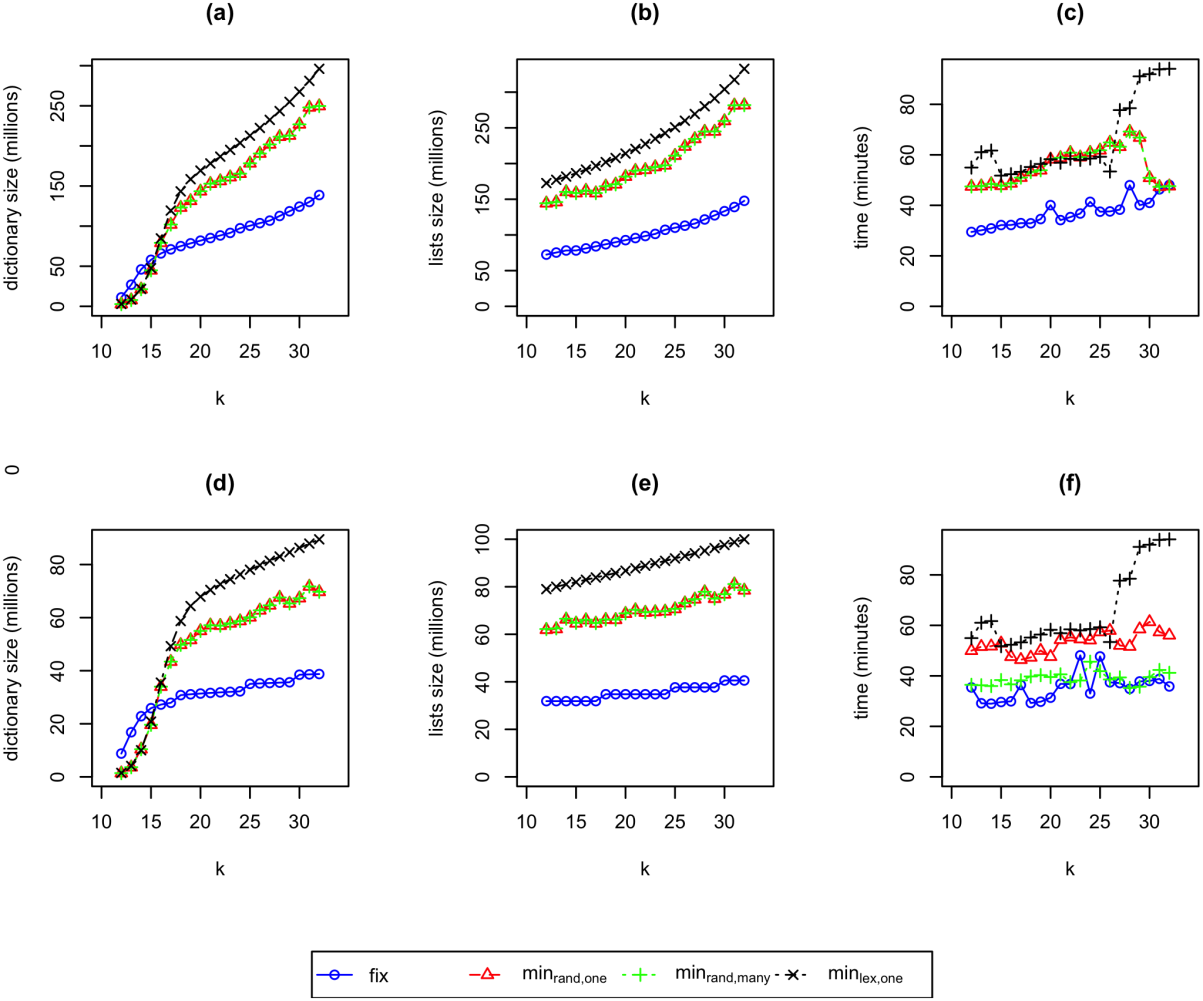
The dictionary sizes, lists sizes, and construction times for *k*-mer indexes built using fixed sampling (*fix*) and minimizer sampling *min*_*rand*,*one*_, *min*_*rand*,*many*_, and *min*_*lex*,*one*_. For parts (a), (b), and (c), we use *L* = 50. For parts (d), (e), and (f), we use *L* = 100. For all graphs, 12 ≤ *k* ≤ 32.

*Fixed sampling* (*fix*) *produces indexes that are less than half the size of those produced by all minimizer sampling methods* (*min*_*rand*,*one*_, *min*_*rand*,*many*_, *and* *min*_*lex*,*one*_) *for almost all choices of* *k*. Likewise, we can construct fixed sampling’s index roughly 1.5 to 1.9 times as fast as we can construct minimizer’s index. We provide full index size and construction time results in Fig 1.

We now explore why fixed sampling produces indexes that are roughly half the size of indexes produced by minimizer sampling *min*_*rand*,*one*_. We start with the size of the occurrence lists. For a fixed value of *k*, we can accurately predict the size of fixed sampling’s *k*-mer occurrence lists because 1/*w* of the total number of *k*-mer occurrences will be sampled. For minimizer, Roberts *et al.* showed that for random sequences, the number of minimizers would be roughly 2/(*w* + 1) of the total number of *k*-mer occurrences [23]. Basically, each minimizer would cover roughly half a window of length *w* rather than a full window of length *w* as we get from fixed sampling. Thus, we would expect fixed sampling to produce occurrence lists that are roughly (*w* + 1)/2*w* the size of the occurrence lists produced by minimizer sampling; that is, the occurrence lists should be just more than half the size. In our experiments, we see that the number of sampled occurrences for fixed sampling divided by the number of sampled occurrences for minimizer sampling ranges from 48% to 55% for both *L* = 50 and *L* = 100 which is consistent with the expectation.

Minimizer sampling *min*_*rand*,*many*_ has essentially identical results to minimizer sampling *min*_*rand*,*one*_ with respect to the size of occurrence lists; the optimization to remove duplicate minimizers from a window does not have much effect on the total number of sampled occurrences. Minimizer sampling *min*_*lex*,*one*_ produced indexes that are larger than minimizer sampling *min*_*rand*,*one*_ with respect to the size of occurrence lists; the optimization to use randomized ordering, instead of lexicographical ordering, effectively reduces over sampling the same *k*-mer in regions with many repeats resulting in 15% to 20% reduction for *L* = 50 and *L* = 100, respectively.

We now consider the dictionary size. For the smallest values of *k* that we consider, mainly 12-15, minimizer typically has much smaller dictionaries than fixed sampling. For *k* = 12 and *L* = 100, minimizer’s dictionary is almost 6 times smaller than fixed sampling’s dictionary. For these small values of *k*, many of the sampled *k*-mers are chosen many times, and this is especially true for minimizer which leads to its smaller dictionary. However, for these *k* values, because many of the sampled *k*-mers are chosen many times, the dictionaries are much smaller than the occurrence lists, so fixed sampling still has a total index size that is roughly half that of minimizer. For example, for *k* = 12 and *L* = 50, for fixed sampling, each *k*-mer in the dictionary appears roughly 6.5 times in the occurrence lists whereas for minimizer sampling *min*_*rand*,*one*_, each *k*-mer in the dictionary appears roughly 52 times in the occurrence lists.

Once we consider *k* ≥ 16, for fixed sampling, each dictionary consists of mostly unique *k*-mers. For example, for *k* = 16 and *L* = 50, each *k*-mer in fixed sampling’s dictionary appears roughly 1.23 times in the occurrence lists. For minimizer sampling *min*_*rand*,*one*_, this starts to happen around *k* = 21. For example for *k* = 21 and *L* = 50, each *k*-mer in minimizer sampling’s dictionary appears roughly 1.24 times in the occurrence lists. By the time *k* = 32, each dictionary *k*-mer appears less than 1.13 times in the occurrence lists for both fixed sampling and minimizer sampling. This implies that for large *k*, the dictionary size is comparable to the occurrence lists size. Specifically, we see that fixed sampling’s dictionaries are roughly half the size of minimizer sampling’s dictionaries for *k* ≥ 21 for both *L* = 50 and *L* = 100. Finally, we note that the dictionary size for minimizer sampling *min*_*rand*,*many*_ is identical to that of minimizer sampling *min*_*rand*,*one*_ as *min*_*rand*,*many*_ only omits some repeated occurrences for the same *k*-mer. Minimizer sampling *min*_*lex*,*one*_ produced dictionaries that are larger than minimizer sampling *min*_*rand*,*one*_, again because we reduce oversampling the same *k*-mer in regions with many repeats. For example, when *k* > 16 the reduction ranges from 12% to 23% for *L* = 50 and *L* = 100, respectively.

We note that for all sampling methods, increasing the value of *k* increases the size of the index. This is expected, since the sampling step *w* = *L* − *k* + 1 decreases as *k* increases.

Finally, fixed sampling’s faster construction time is easily explained. First, the number of sampled occurrences is less than half as many as minimizer sampling. Second, no comparisons are needed; fixed sampling simply grabs every *w*th *k*-mer whereas minimizer sampling needs to consider every *k*-mer and do comparisons to determine if new *k*-mers are minimizers. However, as we show in later, the reduction has significant impact on reducing query time.

### Query time

We first start with our query time results. Our full query time results for each of our three sampling methods for *L* = 50 and *L* = 100 are shown in Tables 4 and 5.

**Table 4:** Query times (in hours) for all sampling methods and all choices of *k* when *L* = 50.

Query set	k	*fix*	*min*_*rand*,*one*_	*min*_*rand*,*many*_	*min*_*lex*,*one*_
Mouse	12	447.00	284.50	437.16	1008.30
	16	106.49	36.08	142.22	204.39
	20	52.91	26.96	107.46	86.13
	24	33.31	17.36	58.38	48.54
	28	20.84	15.48	48.77	28.87
	32	13.75	12.43	32.79	18.58
Chimp	12	1493.09	1294.66	1407.43	2340.63
	16	661.67	510.99	641.86	932.27
	20	340.00	471.84	859.09	455.38
	24	180.67	184.19	221.33	205.94
	28	98.20	116.06	146.56	116.46
	32	55.25	71.34	79.92	56.58
NGS	12	237.04	197.73	205.70	486.43
	16	88.02	79.71	72.66	134.30
	20	41.75	49.40	47.99	59.96
	24	21.33	22.72	22.21	26.69
	28	11.83	14.98	15.73	14.38
	32	6.77	10.68	9.80	7.36

**Table 5:** Query times (in hours) or all sampling methods and all choices of *k* when *L* = 100.

Query set	k	*fix*	*min*_*rand*,*one*_	*min*_*rand*,*many*_	*min*_*lex*,*one*_
Mouse	12	198.46	101.05	102.06	692.17
	16	41.74	5.63	7.25	142.50
	20	21.31	3.59	4.52	40.38
	24	12.37	3.76	4.29	15.60
	28	7.28	2.17	2.48	7.49
	32	4.92	2.16	2.35	4.89
Chimp	12	732.51	524.91	505.38	1744.26
	16	276.38	203.60	203.90	560.79
	20	131.61	160.61	254.88	242.23
	24	61.68	50.29	50.03	98.51
	28	33.20	41.01	40.88	53.60
	32	17.78	30.91	31.53	21.94
NGS	12	111.03	79.95	82.78	364.51
	16	33.33	27.39	28.17	83.38
	20	15.58	13.93	14.47	27.85
	24	7.27	5.91	5.85	10.90
	28	4.12	5.01	5.34	6.08
	32	2.32	4.47	4.43	2.52

*Our key query time result is that for the same* *k* *values and query data with many repeats, such as in the mouse genome*, *min*_*rand*,*one*_ *processes queries significantly faster than fixed sampling, especially for commonly used small* *k* *values like 12 and 16* [4, 17, 44–49]. For example, when *k* = 12 and *k* = 16, *min*_*rand*,*one*_ answer the queries 126.14% and 369.14% faster, on average, than fixed sampling for *L* = 50 and *L* = 100, respectively. For large *k*, *k* > 16, *min*_*rand*,*one*_ processes the queries 58.36% and 271.64% faster, on average, than fixed sampling for *L* = 50 and *L* = 100, respectively. When there are only a few repeats in the query data, such as in the chimp genome and NGS datasets, and for small *k* values, *min*_*rand*,*one*_ is 15.15% to 37.65% faster, on average, than fixed sampling. On the other hand, when the value of *k* > 16, *min*_*rand*,*one*_ is 7.73% to 19.82% slower, on average, than fixed sampling.

While we observe that minimizer sampling process queries faster than fixed sampling for the same choice of *k*, we also observe that minimizer sampling uses more space than fixed sampling for the same choice of *k*. We will later compare minimizer sampling with fixed sampling when they are restricted to indexes of the same size to determine which is indeed faster. When exploring this tradeoff, we find that fixed sampling is faster than minimizer sampling when both methods have equal sized indexes.

*Our next query time result is that increasing* *k* *significantly decreases the query processing time of all methods.* For all query sets, increasing *k* from 12 to 16 reduces the query time of fixed sampling and *min*_*rand*,*one*_ by roughly 3-5 times; the one exception is *min*_*rand*,*one*_ with the mouse genome query set for *L* = 50 and *L* = 100 where the reduction is only 7.89% and 17.96% times, respectively. For all query sets and all methods, increasing *k* by an additive factor of 4 above 16 roughly halves the method’s query processing time with a couple of outliers in both directions.

*Our final query time result is that the optimization using randomized ordering is significantly more effective than the optimization that removes duplicate minimizers, especially for large* *L* *and small* *k* *values*. For the mouse genome and for *k* = 12 and *k* = 16, the randomized ordering is 360.46% to 1508.86% faster, on average, than lexicographical ordering. For the chimp and NGS datasets and *k* = 12 and *k* = 16, the randomized ordering is 81.62% to 280.17% faster, on average, than lexicographical ordering. On the other hand, for the mouse genome *k* = 12 and *k* = 16, duplicate minimizer removal is only 14.94% to 173.93% faster, on average, than standard minimizer sampling without duplicate removal. For the chimp and NGS datasets, duplicate minimizer removal does not improve minimizer sampling’s query time; the one exception is for chimp data when *L* = 100 and *k* = 12 and *k* = 16 where duplicate removal is 17.16% faster, on average, than standard minimizer without duplicate removal.

#### The theoretical vs. empirical query time expectation

Because minimizer sampling only tests some *k*-mers extracted from the query sequence to see if they are shared *k*-mers, one might expect that minimizer sampling would process queries as much as *w* times faster than fixed sampling. However, the query time results show this is not the case; the speedup is typically much less than *w* and often less than *twice* as fast. This is explained by counting the total number of shared *k*-mer occurrences found by both fixed and *min*_*rand*,*one*_. These counts are shown in Tables 6 and 7.

**Table 6:** The number of shared *k*-mer occurrences (in billions) for all sampling methods and all choices of *k* when *L* = 50.

Query set	k	*fix*	*min*_*rand*,*one*_	*min*_*rand*,*many*_	*min*_*lex*,*one*_
Mouse	12	281.90	100.58	348.92	870.72
	16	103.19	25.02	202.80	242.16
	20	55.96	20.84	161.16	101.93
	24	33.28	11.33	83.47	52.93
	28	19.55	11.68	73.18	29.27
	32	11.74	9.36	47.14	16.98
Chimp	12	714.46	445.52	515.02	1699.86
	16	310.33	176.65	223.43	601.94
	20	155.08	255.68	520.61	221.16
	24	75.52	62.58	85.37	87.17
	28	37.92	46.97	109.50	45.74
	32	22.12	27.64	45.33	23.79
NGS	12	101.81	64.62	71.33	390.05
	16	30.29	24.05	26.56	86.30
	20	12.95	17.20	20.26	28.24
	24	6.41	7.24	8.24	11.00
	28	3.44	5.11	5.95	5.86
	32	2.09	3.42	3.91	3.06

**Table 7:** The number of shared *k*-mer occurrences (in billions) for all sampling methods and all choices of *k* when *L* = 100.

Query set	k	*fix*	*min*_*rand*,*one*_	*min*_*rand*,*many*_	*min*_*lex*,*one*_
Mouse	12	124.51	25.91	35.00	663.35
	16	40.72	1.49	4.24	156.26
	20	20.98	0.69	2.33	44.94
	24	10.94	0.57	1.27	13.70
	28	6.04	0.10	0.36	5.09
	32	3.25	0.10	0.50	2.82
Chimp	12	355.18	157.24	160.65	1499.97
	16	129.05	61.04	61.30	418.83
	20	58.20	87.84	174.00	125.58
	24	24.31	13.01	13.05	37.12
	28	11.72	10.77	11.62	15.88
	32	6.08	8.09	8.10	6.35
NGS	12	44.95	22.98	23.41	318.71
	16	11.89	7.81	7.86	59.64
	20	4.86	4.67	4.71	14.79
	24	2.08	1.54	1.55	4.17
	28	1.06	1.36	1.37	1.82
	32	0.58	1.09	1.10	0.73

Recall fixed sampling will test all *q* − *k* + 1 *k*-mers from a query sequence *q*. On the other hand, the expected number of query *k*-mers that minimizer sampling will test is 2(*q* − *k* + 1)/(*w* + 1) or roughly 2/(*w* + 1) times smaller if the sequences are generated uniformly at random [23]. Each tested *k*-mer will generate *x* shared *k*-mer occurrences where *x* is the length of that *k*-mer’s occurrence list in the index. Theoretically, if *x* = *c* for fixed sampling, then we expect that *x* = 2*c* for minimizer sampling. Then fixed sampling will test *c*(*q* − *k* + 1) *k*-mers occurrences and minimizer sampling will test 2*c*(*q* − *k* + 1)/(*w* + 1) of *k*-mers occurrences; since minimizer sampling produce lists large by a factor of two. However, this is not always the case. For example, for *q* = 1000 and *L* = 100, then we expect minimizer sampling to be 95% faster than fixed sampling when 20 ≤ *k* ≤ 32. For chimp and NGS datasets, the empirical results show that minimizer sampling is 7.73% to 14.24% slower than fixed sampling.

To understand the query time, we need to compute the average size of *x* for *k*-mers that are in the index dictionary. We show this in Tables 8 and 9 for each sampling method; specifically, these tables show the mean and the standard deviation of occurrence list lengths in each index. For *L* = 100 and *k* = 12, the mean length of a minimizer sampling occurrence list is just over 13 times larger than the mean length of a fixed sampling occurrence list. For *k* = 16, this falls to roughly 1.55 times larger, and for larger *k*, this falls to just a bit larger. Even more dramatic, the standard deviation for minimizer’s occurrence list lengths ranges from 6.58 times up to 21.5 times larger than the standard deviation of fixed samplings occurrence list lengths.

**Table 8:** The mean and standard deviation of the length of a *k*-mer’s list of occurrences using the human genome for all sampling methods and all choices of *k* when *L* = 50.

	k	*fix*	*min*_*rand*,*one*_	*min*_*rand*,*many*_	*min*_*lex*,*one*_
Mean	12	6.50	51.72	53.20	58.70
	16	1.23	2.01	2.05	2.26
	20	1.13	1.26	1.27	1.26
	24	1.10	1.19	1.19	1.19
	28	1.08	1.16	1.16	1.15
	32	1.07	1.13	1.13	1.12
Std. Dev.	12	37.61	419.57	483.85	616.48
	16	10.17	67.30	66.02	69.38
	20	6.65	32.14	36.96	32.79
	24	4.66	20.06	22.73	19.98
	28	3.41	17.56	16.91	13.74
	32	2.65	10.61	11.84	9.09

**Table 9:** The mean and standard deviation of the length of a *k*-mer’s list of occurrences using the human genome for all sampling methods and all choices of *k* when *L* = 100.

	k	*fix*	*min*_*rand*,*one*_	*min*_*rand*,*many*_	*min*_*lex*,*one*_
Mean	12	3.63	45.20	44.70	53.02
	16	1.17	1.81	1.95	2.34
	20	1.11	1.24	1.25	1.28
	24	1.08	1.19	1.19	1.19
	28	1.06	1.16	1.15	1.15
	32	1.05	1.13	1.13	1.12
Std. Dev.	12	18.68	401.81	402.42	712.16
	16	6.21	67.08	61.54	80.71
	20	4.02	25.38	30.99	36.05
	24	2.62	17.51	19.63	20.47
	28	1.87	16.71	14.84	13.85
	32	1.37	9.02	10.11	8.61

What this shows is that some *k*-mers in minimizer sampling have very large occurrence lists. Furthermore, the *k*-mers that have large occurrence lists are exactly the *k*-mers that are most likely to be extracted from a query sequence since the sampling method is biased to choose them. This explains why, despite testing relatively few query *k*-mers, minimizer sampling have much larger query times than expected theoretically.

### Space and speed

**Fig 2:**
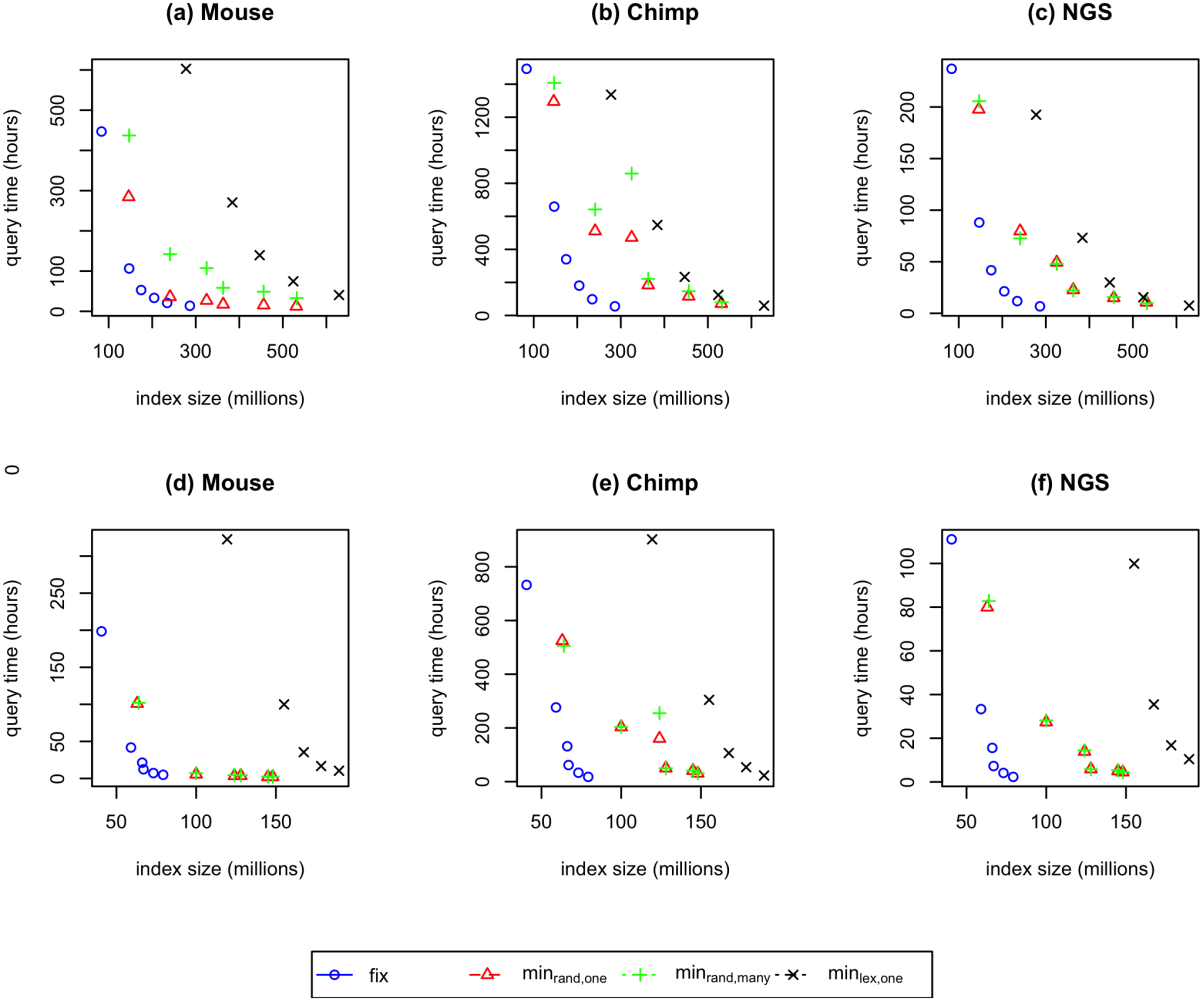
Comparing the space and speed of fixed sampling (*fix*), minimizer sampling *min*_*rand*,*one*_, *min*_*rand*,*many*_, and *min*_*lex*,*one*_. We use *L* = 50 for the three upper figures and *L* = 100 for the three lower figures. The values for *min*_*lex*,*one*_ when *k* = 12 are very large and removed from figure below.

We summarize our comparison of the the sampling methods by plotting the space and speed of the resulting index for each query set and both choices of *L* in Fig 2.

If we ask both methods to use the same space regardless of *k*, we find that fixed sampling typically answers queries at least as fast as *min*_*rand*,*one*_ and often is faster. For example, the index created using fixed sampling with *k* = 16 has roughly the same size as the index created using minimizer sampling with *k* = 12. However, fixed sampling is 37.43%, 51.11%, and 44.52% faster than minimizer sampling for the mouse, chimp, and NGS datasets, respectively. Combined with the fact that fixed sampling is much simpler than minimizer sampling, fixed sampling is always the best choice if we can choose *k* to optimize both time and space.

Finally, we observe that the randomized ordering optimization is more effective than the duplicate removal optimization. We can see that for all *k* values we consider, the effectiveness of *min*_*rand*,*many*_ and *min*_*rand*,*one*_ are very similar. On the other hand, *min*_*lex*,*one*_ is significantly worse than both *min*_*rand*,*one*_ and *min*_*rand*,*many*_.

## Conclusion

We now summarize our main conclusions. When comparing fixed and minimizer sampling, our results show that if we use the same *k*-mer size, minimizer sampling is only slightly faster than fixed sampling, despite sampling from the query sequence. If we are allowed to use any *k*-mer size for each method, then we can choose a *k*-mer size such that fixed sampling both uses less space and processes queries faster than minimizer sampling. As is common in many applications, there is a space versus speed tradeoff. Using a larger *k* requires more space but results in smaller query times. The key benefit of increasing *k* is that there are many fewer false positives which leads to much faster query processing. On average, the reduction in query times for all query sets when *k* = 32 compared to *k* = 12 is 37 and 136 times faster for fixed sampling and minimizer sampling, respectively.
